# Efficacy of an XPO1 inhibitor in combination with irinotecan in a preclinical colorectal cancer model

**DOI:** 10.3389/fonc.2026.1721685

**Published:** 2026-03-31

**Authors:** Robert W. Lentz, Adrian T. A. Dominguez, Nicole Baranda Balmaceda, John J. Arcaroli, Stacey M. Bagby, Cameron A. Binns, Stephen G. Smoots, Marilyn M. Jackson, Morgan Macbeth, Tessa Holmstoen, Tapahsama Banerjee, Phaedra A. Whitty, S. Stephen Yi, Christopher H. Lieu, Wells A. Messersmith, Todd M. Pitts

**Affiliations:** 1 Division of Medical Oncology, Department of Medicine, University of Colorado School of Medicine, Aurora, CO, United States; 2 Division of Cancer Medicine, Department of Gastrointestinal Medical Oncology, University of Texas MD Anderson Cancer Center, Houston, TX, United States; 3 Baylor Research Institute, School of Medicine-Temple, Baylor College of Medicine, Temple, TX, United States

**Keywords:** chemotherapy, colorectal cancer, eltanexor, selinexor, XPO1

## Abstract

**Background:**

Irinotecan is a topoisomerase I inhibitor that is commonly used as a chemotherapeutic regimen for the treatment of metastatic colorectal cancer (CRC) by producing cytotoxic DNA damage. However, drug resistance frequently occurs, underscoring the importance of developing newer treatment strategies. The nuclear export protein XPO1 is an important mediator in the transport of proteins involved in cell cycle regulation and XPO1 inhibitors have shown activity as a single agent and in combination with DNA damaging agents.

**Methods:**

Using preclinical CRC PDX and cell line models, we evaluated the therapeutic potential of selinexor or eltanexor (XPO1 inhibitors), as a single agent and in combination with chemotherapeutic agents.

**Results:**

We show a combination effect with eltanexor + irinotecan in one of our CRC PDX models and observed synergistic sequential treatment effects in three of our CRC cell lines. In our mechanistic studies, elevation of p53 nuclear/cytoplasmic ratio and the activation of H2A.X suggests that the addition of eltanexor to irinotecan may facilitate its cellular effects by preventing the repair of DNA damage and inducing apoptosis.

**Conclusion:**

This preclinical CRC study demonstrates that combining eltanexor with irinotecan enhances cytotoxicity and anti-tumor effects in a subset of preclinical CRC PDX and cell line models, and that sequential treatment with irinotecan first may be most effective in inducing these treatment effects in the combination responsive CRC models.

## Introduction

Colorectal cancer (CRC) is a common and deadly disease. While improved screening and treatments have lengthened survival, the 5-year survival probability of metastatic CRC (mCRC) is only 13% (1). First- and second-line standard of care treatments for microsatellite stable (MSS) mCRC are cytotoxic chemotherapy [combinations of oxaliplatin, irinotecan, and fluoropyrimidines] with or without inhibitors of epidermal growth factor receptor (EGFR) and vascular endothelial growth factor (VEGF) (2). Still, the duration of response to these treatments is limited. In the second-line, progression-free survival with an irinotecan-containing regimen is only six months (3, 4). Further investigation into mechanisms of topoisomerase-I inhibitor resistance and additional treatment strategies for MSS mCRC are desperately needed.

Exportin 1 (XPO1) is the major nuclear export protein for tumor suppressor proteins (TSPs), cell cycle regulators, and oncoproteins (5–7). Overexpression is seen in many cancer types, including CRC, and may portend a worse prognosis (6–8). XPO1 inhibition has been recognized as an appealing cancer treatment strategy, resulting in nuclear localization of TSPs (activating cell cycle arrest), oncoprotein mRNAs, and topoisomerases, and decreases the DNA damage repair (DDR) response, ultimately resulting in apoptosis (5, 6, 9, 10).

Selinexor (KPT-330) and eltanexor (KPT-8602) are selective inhibitors of nuclear export (SINE) that bind XPO1 in a reversible and selective fashion (11–13). Selinexor monotherapy has been evaluated pre-clinically in multiple solid tumor types (8, 14–19). While cell line and patient-derived xenograft (PDX) data support anti-cancer efficacy of selinexor monotherapy, combination therapy appears to be more efficacious and may reverse drug resistance. Interestingly, administering DNA damaging agents followed by XPO inhibition results in synergistic cytotoxicity compared to concurrent chemotherapy and XPO1 inhibition or XPO1 inhibition followed by cytotoxic therapy (10, 20).

In colon cancer cell lines, combining the SINE KPT-185 with SN38 (active metabolite of irinotecan) synergized to increase apoptosis and cell cycle arrest (18). In irinotecan/SN38-resistant and -sensitive CRC cell lines, another SINE, KPT-251, demonstrated synergy with SN38 *in vitro* and irinotecan *in vivo* by localizing topoisomerase I to the nucleus (21). In a CRC murine xenograft, the SINE KPT-276 increased apoptosis, decreased Ki67, upregulated nuclear localization of p53 and p21, and inhibited tumor growth (19). The utility of XPO1 inhibition in the mCRC clinical setting is not fully understood.

Similar to *in vitro* studies, early phase clinical trials with single agent selinexor have been limited with suboptimal response rates in patients with mCRC (19). In a phase I dose-escalation clinical trial combining mFOLFOX (fluorouracil, leucovorin, and oxaliplatin) and selinexor, dose-limiting toxicities were common and limited efficacy assessment (22).

To allow for better tolerability, eltanexor, an investigational second-generation XPO1 inhibitor was developed. Eltanexor has reduced penetration across the blood-brain barrier compared to selinexor, allowing for more frequent dosing and better tolerability *in vivo* (13). In a phase 1 clinical trial of eltanexor in patients with relapsed/refractory multiple myeloma, eltanexor appeared to be better tolerated and potentially more effective than selinexor in cross trial comparison with selinexor (23).

To further understand the role of XPO1 inhibition in mCRC, we evaluated the combination of the XPO1 inhibitor selinexor with irinotecan and 5-FU in one CRC PDX model and eltanexor with irinotecan/SN38 in 2 CRC PDX models and 4 CRC cell lines. We hypothesized that XPO1 inhibition may improve response to topoisomerase-I inhibition in mCRC by impairing the DDR response.

## Materials and methods

### Chemicals and reagents

Eltanexor (KPT-330) and selinexor were obtained from Karyopharm Therapeutics. SN38 (S4908) was purchased from Selleck Chem, and irinotecan was purchased from the University of Colorado Research Pharmacy. Selinexor was made weekly in a vehicle of 0.6% Poloxamer Pluroniv F-68 and 0.6% Plasdone PVP K-29/32 in HPLC H H_2_O (vortexing 30 seconds and sonicating for ~15 minutes until dissolved, stored at room temperature protected from light). Eltanexor was made daily in a vehicle of 1% tween80 and 0.5% methylcellulose in HPLC H_2_O (vortexing 30 seconds or until dissolved, sonicating 1 hour, and stirring overnight at 4 °C). Irinotecan was made fresh each week, diluted in sterile saline to reach the concentration needed for mice. All antibodies for western blot and immunocytochemistry (ICC) were acquired from Cell Signaling Technologies: RAD51 Paralog B (RAD51) (#8875), H2A histone family member X (p -H2A.X) (#9718), MutS Homolog 2 (MSH2) (#2017), TP53 (p53) (#2527), cyclin-dependent kinase inhibitor 1A (CDKN1A/p21) (#2946), GAPDH (#2118), Laminn A/C (4777), p-H2A.X (Alexa Fluor^®^ 647 Conjugate) (#9720), p53 (7F5) Rabbit mAb (Alexa Fluor^®^ 488 Conjugate) (#5429), p21 Waf1/Cip1 (12D1) Rabbit mAb (Alexa Fluor^®^ 647 Conjugate) #8587 goat anti-rabbit IgG (H+L) antibody (#35401), anti-mouse IgG (H+L), and F(ab’)2 fragment (Alexa Fluor^®^ 647 Conjugate) (#4410).

### Patient-derived xenograft study

Patient-derived tumor tissues were collected from patients pathologically diagnosed with CRC under the approval of the Colorado Multiple Institutional Review Board. In this study, we evaluated 3 different CRC patient derived tumor models. Approximately 6–8-week-old athymic nude mice (Inotiv, Athymic Nude-Foxn1nu, MGI:5652489) were anesthetized with 5% isoflurane and maintained at 1-2% using a vaporizer. They were then injected subcutaneously into both flanks as described previously (24). Animals were treated with eltanexor or selinexor, irinotecan or irinotecan + 5-fluorouracil (5FU), eltanexor + irinotecan or selinexor + irinotecan + 5FU once tumor volumes reached ~ 100 – 300 mm^3^ with a group average between ~ 180 – 265 mm^3^. CRC254 experiment 1 eltanexor dosing was 10 mg/kg PO daily Monday thru Friday initially for eight days, followed by a reduction to 7.5 mg/kg; or CRC254 experiment 2: 5mg/kg PO daily Monday thru Friday and irinotecan was 15 mg/kg Intraperitoneal (IP) once weekly. BPB56DDD experiments selinexor dosing was 10 mg/kg PO 3 times per week (Monday/Wednesday/Friday) initially for 12 days, followed by a reduction to 2 times per week (Monday/Thursday), irinotecan was 15 mg/kg IP once weekly, and 5FU was 60 mg/kg IP once weekly. CRC238 experiment eltanexor dosing was 5 mg/kg PO daily Monday thru Friday and irinotecan was 15 mg/kg IP once weekly. CRC254 experiment 1 had 14-15 tumors per group and CRC254 experiment 2 had 8 tumors per group. BPB56DDD experiment had 12 tumors per group and CRC238 had 9 tumors per group. Mice weights and tumor measurements were collected twice per week, health was evaluated daily and were humanely euthanized by anesthetizing with 5% isoflurane for a deep plane of anesthesia followed by cervical dislocation at either experimental, tumor, or health endpoints. All animal studies were done in accordance with University of Colorado Institutional Animal Care and Use Committee. **Table 1** shows mutations and microsatellite status of the CRC cell lines and PDX.

**Table 1:** Mutation and microsatellite status of CRC Models.

PDX/cell Line	*KRAS*	*TP53*	*PIK3CA*	*CTNNB1*	Microsatellite status
PDX					
BPB56DDD	UK	UK	UK	UK	MSS
CRC254	WT^2^	P72R^2^	K51R, S66T, I391M^2^	WT^2^	MSS
CR238	Q61K^2^	P72R^2^	WT^2^	S246P^2^	MSI-H
Cell line					
HCT116	G13D^1,2^	WT^2^	H1047R^1,2^	S45del^1,2^	MSI-H
HCT15	G13D^1,2^	S241F^1,2^	E545K, D549N^1,2^	WT^2^	MSI-H
HCT8	G13D^2^	WT^2^	WT^2^	WT^2^	MSI-H
LS1034	A146T^1,2^	G245S^1,2^	WT^2^	WT^2^	MSS

MSI-H, microsatellite instability-high; MSS, microsatellite stable; WT, wild-type; UK, Unknown.

1. Cancer Cell Line Encyclopedia (CCLE) https://sites.broadinstitute.org/ccle/.

2. In house RNA Seq and/or exome sequencing.

### RNA isolation and qRT-PCR

Tumor tissues (vehicle and treated) from mice were homogenized using a Qiagen TissueLyser. The total RNA of the tumor tissue was isolated using the Qiagen RNeasy Mini Kit (#74106) according to the manufacturer’s instructions. Quantitative real-time PCR was performed using Taqman primer probes MutL Homolog 1 (*MLH1)* (Hs00179866), (*MSH2)* (Hs00953523), MutS Homolog 3 (*MSH3)* (00989003), Beta-Glucuronidase (*GUSB)* (Hs99999908), *p53* (Hs01034249), RAD50 Double Strand Break Repair Protein (*RAD50)* (Hs00990023), (*RAD51B)* (Hs00172522), Exportin 1 (*XPO1)* (Hs00185645), Glyceraldehyde-3-Phosphate Dehydrogenase (*GAPDH)* (02786624), and TaqMan Master Mix (Applied Biosystems #4444556). Analysis was performed using Quant Studio 3 system and the DA2 Software (Applied Biosystems #4304437). Data was normalized to *GAPDH* levels and were presented as mean ± standard error of the mean (SEM), and significance was calculated by a one-way ANOVA. Results are the mean of triplicate measurements.

### Cell culture and viability assay

Human CRC cell lines HCT8 (RRID: CVCL_2478), HCT15 (RRID: CVCL_0292), HCT116 (RRID: CVCL_0291), and LS1034 (RRID: CVCL_1382) were purchased from American Type Culture Collection (ATCC) (Manassas, VA; **Table 1**). Cells were grown in Dulbecco’s Modified Eagle Medium (DMEM) with 10% fetal bovine serum, penicillin/streptomycin, and non-essential amino acids. Cell lines were maintained at 37 °C containing 5% CO_2_. Cell lines were all authenticated and tested for mycoplasma prior to use. Viability assay was conducted by plating 1x10^4^ adherent cells in each well of a white-walled tissue-treated 96-well plate. Cells were treated concurrently or sequentially for 72 hours. For sequential treatment, the CRC cell lines were treated with SN38 (0- 30 nM) for 24 hours, washed with PBS, and then treated with eltanexor (0-100 μM) for an additional 48 hours. Cells were then treated with CellTiter-Glo 2.0 Cell Viability Assay reagent (Promega #G9243), and viability was measured by luminescence. Results are the mean of triplicate measurements.

### Immunoblotting

Proteins were prepared using RIPA Lysis Extraction Buffer (Thermo Scientific, #89900) with the addition of protease and phosphatase inhibitors (Thermo Scientific, #1861282). Protein concentration was quantified using the Bicinchoninic acid (BCA) protein assay kit (Pierce #A55864). Equal amounts of proteins were subjected to NuPAGE 4-12% BisTris gel and transferred onto polyvinylidene fluoride (PVDF) membranes. Membranes were blocked in Blocking Buffer (TBS 0.1% Casin) for 1 hour at room temperature, followed by overnight incubation with primary antibody (1:1000) at 4°C. Membranes were washed and then incubated with secondary primary conjugated fluorescent antibodies (1:10,000) for 1 hour at room temperature. Using the fluorescent channels 600nm and 800nm, proteins were detected, and images were captured using the Odyssey Infrared Imaging System (Li-Cor).

### Nuclear isolation

HCT8 and HCT116 were plated 2x10^6^ cells in 100 mm culture dishes and allowed to adhere overnight. Then, SN38 [10 nM] and eltanexor [1 uM] were added for 12 hours and nuclear extraction was performed according to manufacturer’s instructions of the kit (Thermo, 7833).

### Immunocytochemistry

Approximately 1x10^4^ adherent cells were plated in each well of the Nunc Lab-Tek II Chamber Slide System (#154534). Wells were incubated at different time points. For staggered time points of SN38, cells were washed and then eltanexor was incubated for an additional 48 hours. Single-agent SN38 conditions were allowed to recover for the same 48 hours as the combination treatments. Cells were then fixed with 4% paraformaldehyde for 15 minutes, washed with PBS, and then permeabilized using PBS with 0.1% Triton X-100 for 20 minutes. Cells were blocked for 30 minutes in PBS with 5% goat serum, followed by primary antibody incubation [P-H2A.X (#9720) 1:500, p53 (#5429) 1:100, p21 (#8587) 1:300] at 4°C overnight. Cells were then washed 3 times and incubated with a secondary antibody for 1 hour at room temperature. Cells were washed 3 times and then DAPI was added (1 mg/mL) for 5 minutes, washed, and mounted using an antifading mounting medium. 20x images were captured on an Olympus IX83 microscope and CellSens Software.

### Statistical analysis

A Brown-Forsythe and Welch ANOVA tests and Unpaired t test with Welch’s correction with the individual variances computed for each comparison were performed at the end of treatment to determine whether there were significant differences among all treatment groups by Specific Growth Rate [SGR = ln(Volume of tumor final/Volume of tumor initial)/(Treatment end day - Treatment start day)]. The mean is shown with each having a bar indicating the SE of the mean. Data analyses were performed with GraphPad Prism statistical software (RRID: SCR_002798, GraphPad, San Diego). Bliss synergy score was analyzed using SynergyFinder and is defined as Bliss Score = (%inhibition of combination treatment)-(Sum of %Inhibition of single agents).

## Results

### Assessment of treatment effects of selinexor or eltanexor and in combination with chemotherapy in our CRC PDX models

**Figure 1:**
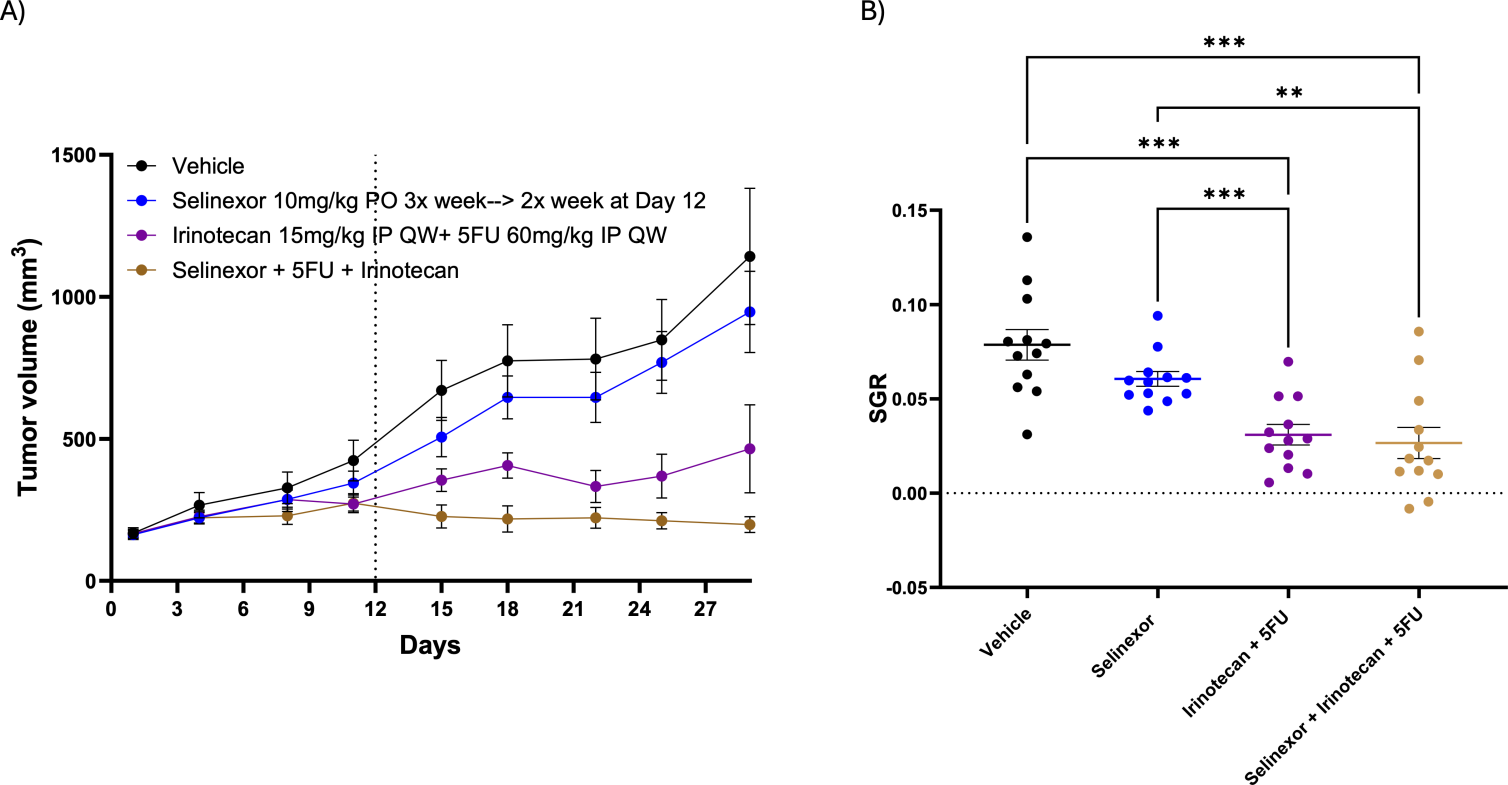
Patient derived xenograft (PDX) tumor BPB56DDD treated with single agent selinexor, irinotecan + 5FU or triple combination. **(A, B)** Assessment of tumor volumes **(A)** and specific growth rate (SGR) **(B)** after 29 days of treatment (10 mg/kg 3 x week reduced to 2 x week on day 12 due to apparent toxicity). **p<0.01, ***p<0.001.

**Figure 2:**
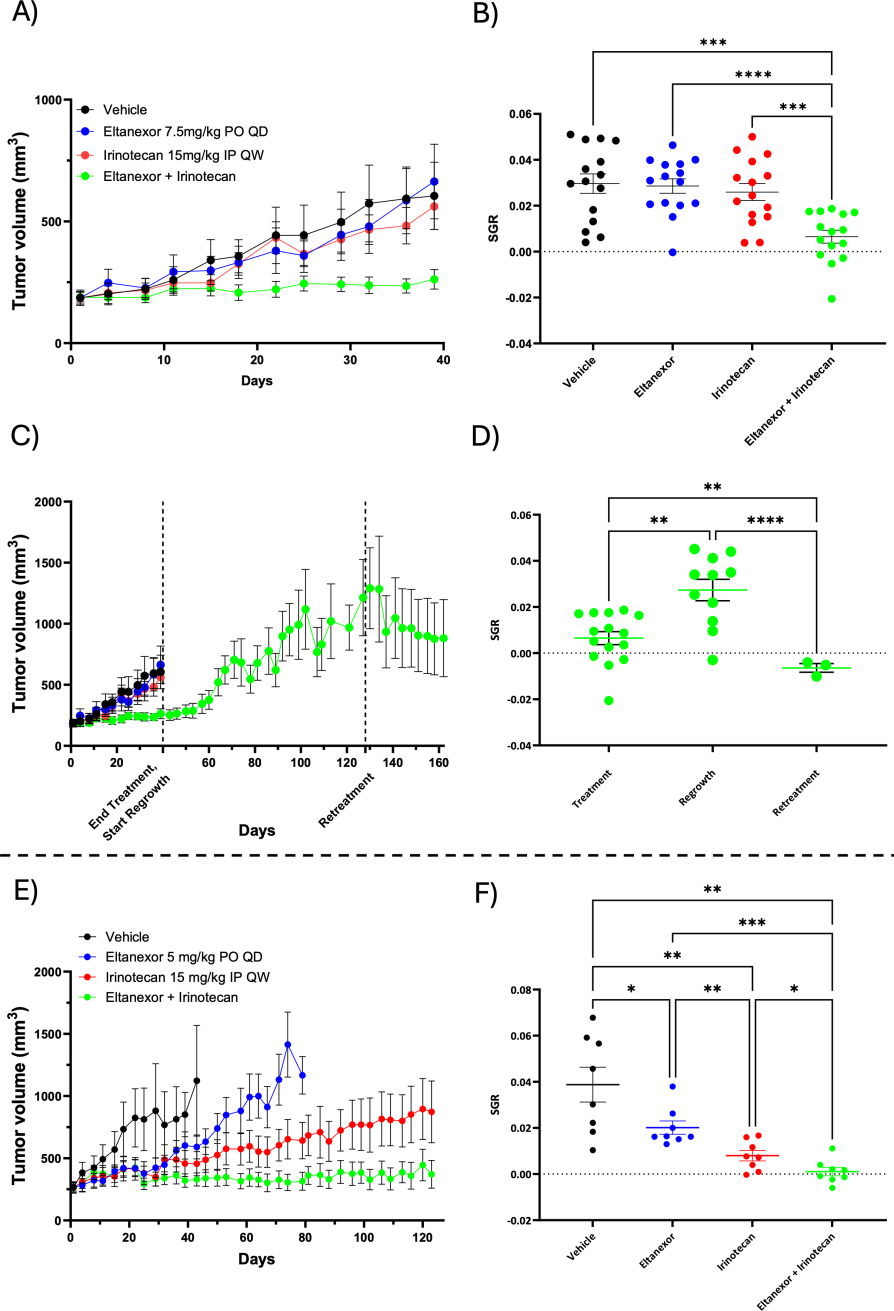
Patient derived xenograft (PDX) tumor CRC254 treated with single agent eltanexor, irinotecan or combination. A and B) Assessment of tumor volumes **(A)** and specific growth rate (SGR) **(B)** after 39 days of treatment (10 mg/kg reduced to 7.5 mg/kg on day 8 due to toxicity). **(C, D)** Evaluation of tumor volumes **(C)** and SGR **(D)** (treatment, regrowth, and retreatment) in combination (eltanexor 7.5 mg/kg + irinotecan 15 mg/kg) treated tumors (continuation of the combination experiment in **Figure 1A**). In this experiment, combination treatment was stopped at day 39, and combination tumors were allowed to regrow for 98 days, followed by resumption of combination treatment for another 34 days (end of treatment 162 days). **(E, F)** Treatment of CRC 254 with a reduced eltanexor dose of [5 mg/kg] and/or irinotecan [15 mg/kg]. Evaluation of tumor volumes **(E, F)** SGR after 120 days of treatment. *p<0.05, **p<0.01, ***p<0.001, ****p<0.0001.

We first examined the effects of the XPO1 inhibitor selinexor in combination with irinotecan and 5-fluoruracil (5-FU) in our CRC PDX model BPB56DDD. Treatment with single agent selinexor did not result in a significant reduction in tumor growth when compared to mice treated with vehicle (**Figure 1**). A significant decrease in tumor growth was observed in mice treated with irinotecan + 5-FU and with the triple combination selinexor + irinotecan + 5-FU when compared to vehicle and selinexor treated groups; however, there were no significant differences between irinotecan + 5-FU *vs*. selinexor + irinotecan + 5-FU. (**Figure 1**). Next, we assessed the effects of the XPO1 inhibitor eltanexor in our CRC PDX model (CRC254), as the company decided to prioritize this next generation XPO1 inhibitor. Mice were treated with eltanexor, irinotecan, or the combination for 39 days. As depicted in **Figure 2A** (tumor volume) **Figure 2B** (SGR), no difference with eltanexor (10 mg/kg PO daily Monday-Friday, decreased to 7.5 mg/kg on day 8 due to toxicity as discussed below) or irinotecan (15 mg/kg weekly) single agent therapy was observed when compared to vehicle. In contrast, the combination of eltanexor + irinotecan significantly reduced tumor growth over 39 days of treatment (**Figure 2A, B**). Of note, no significant difference in body weights were seen across all groups treated over the 39-day treatment, but the combination treatment did experience a much more prominent weekly up and down in weights, contributing to a long-term decrease in health (**Supplementary Figures 1A, C**). In the first 8 days of treatment with eltanexor 10 mg/kg, we noted a 5-10% decrease in net body weight and observed the start of adverse health effects such as mild to moderate lowering body condition, cooler temperature, and lethargy. Hence, based off this mild decrease in weights, observable adverse health, and previous work with selinexor toxicities, we lowered the eltanexor dose to 7.5 mg/kg to prevent a further decline in health. A key finding here is that we were still able to orally dose once per day with eltanexor as opposed to selinexor which was reduced to twice per week oral dosing because of more severe toxicities seen (data not shown or will be in supplemental)??. Next, we evaluated the effects after stopping treatment at day 39 and rechallenging combination treatment after 89 days, where we retreated mice for another 34 days (end of treatment 162 days). **Figures 2C, D** show that discontinuation of combination therapy (end treatment day 39) significantly enhanced tumor growth while retreatment with eltanexor + irinotecan at day 128 resulted in a decrease in tumor growth when compared to end of study treatment day 39 and at regrowth day 128. Retreatment of combination therapy at day 128 again showed similar toxicities as the initial treatment in the first 8 days, where one mouse did eventually recover weight and the other did not (**Supplementary Figures 1E, F**). Given the moderate challenge in tolerability with the 7.5 mg/kg eltanexor + irinotecan treatment (especially in the first 8 days of treatment), we performed a second study (CRC254) where we tested eltanexor at a dose of 5 mg/kg as a single agent and in combination with irinotecan with a treatment duration of 123 days. As shown in **Figures 2E, F**, single agent eltanexor and irinotecan both decreased SGR compared to vehicle, and combination treatment further decreased SGR compared to either single agent. The 5 mg/kg daily oral dose used in this experiment was well-tolerated, as shown by an initial decrease in weights but recovery and increase in body weights in all treatment groups and very few observable adverse health effects noted (**Supplementary Figures 1B, D**). Finally, we treated one additional CRC PDX (CRC238) with eltanexor, irinotecan, and eltanexor + irinotecan for 23 days. In this CRC PDX model, single agent eltanexor at a dose of 5mg/kg as well as irinotecan and eltanexor + irinotecan significantly reduced tumor growth when compared to vehicle. However, we did not observe a combination effect that resulted in a further decrease in tumor growth when compared to single agent eltanexor or irinotecan. (**Supplementary Figure 2**). These results demonstrate that differential treatment effects were seen with the XPO1 inhibitors selinexor or eltanexor as a single agent and in combination with chemotherapy in different CRC models.

### Examination of DNA mismatch repair genes, (GUSB) and XPO1 expression in our treated CRC PDX

**Figure 3:**
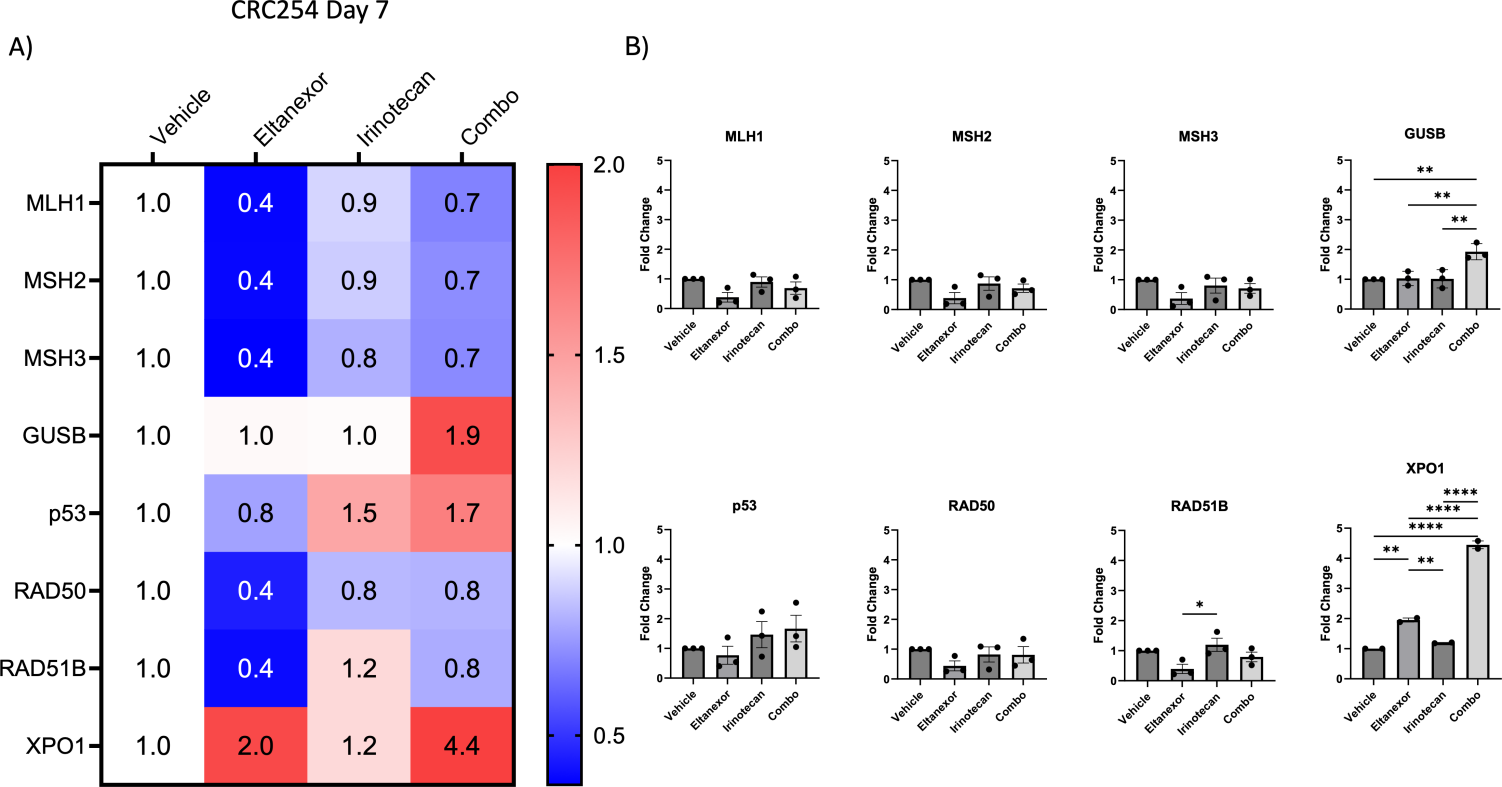
Examination of tumor gene expression of mismatch repair genes, GUSB and XPO1 in our CRC254 patient derived xenograft. Following 7 days of treatment, RNA was isolated and quantitative polymerase chain reaction (qPCR) was performed to evaluate treatment effects (eltanexor [5mg/kg], irinotecan [15mg/kg] or combination) on gene expression **(A)** Heatmap and **(B)** bar graphs of the gene expression profiles after 7 days of treatment in mismatch repair genes, GUSB and XPO1. *p<0.05, **p<0.01, ****p<0.0001.

Irinotecan treatment has several antitumor effects including facilitating double-strand DNA breaks (25); therefore, we assessed the effects of eltanexor, irinotecan, and in combination on the DNA pathway repair genes *MLH1*, *MSH2*, *MSH3*, *p53*, *RAD50*, and *RAD51B*. Gene expression levels were evaluated by RT-PCR in eltanexor-treated (5 mg/kg) CRC254 tumors at day 7, normalized to vehicle expression (corresponding to **Figure 1E**). As shown in **Figure 3A, B**, there were no differences among all treatment groups with respect to the mismatch repair genes *MLH1*, *MSH2*, *MSH3*, and *RAD50* at the time point examined. An increase in *p53* gene expression was seen in the irinotecan and combination group; however, this was not statistically significant. *RAD51B* was significantly higher in the irinotecan-treated group *vs*. eltanexor. In addition, beta-glucuronidase (*GUSB*) was significantly higher in the combination group when compared to vehicle, eltanexor, and irinotecan. Finally, we determined whether treatment altered *XPO1* gene expression after 7 days of treatment. While eltanexor elevated *XPO1* gene expression when compared to vehicle and irinotecan, the greatest increase in *XPO1* expression was seen in the combination treatment group (**Figures 3A, B**). These results demonstrate that, *in vivo*, eltanexor exhibits on-target effect as demonstrated by induction of *XPO1* gene expression.

### The effects of concurrent *vs* sequential treatment in CRC cell lines

**Figure 4:**
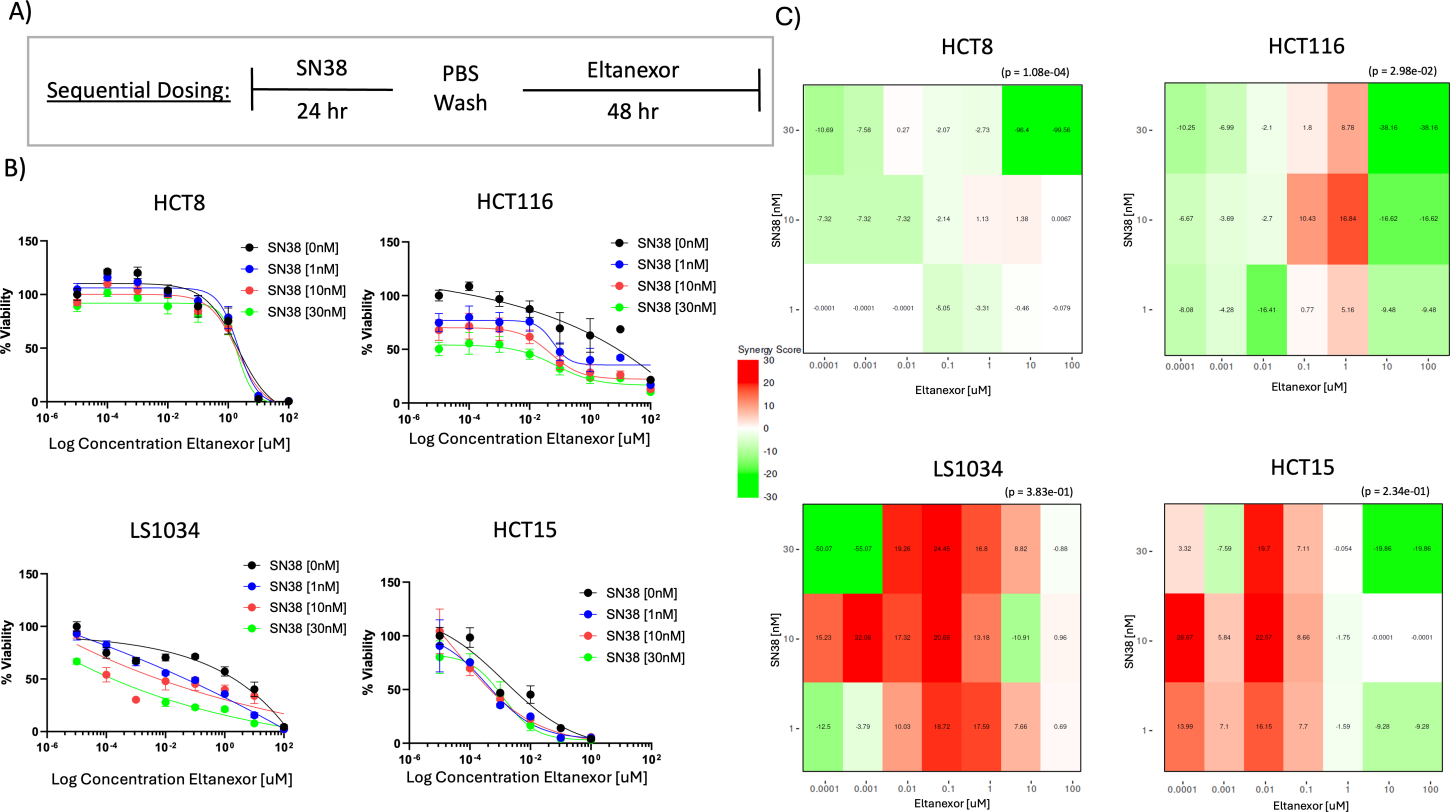
*In vitro* evaluation of sequential dosing of SN38 and/or eltanexor in CRC cell lines (HCT8, HCT116, LS1034, and HCT15). **(A)** Dosing strategies for *in vitro* viability assay. Cells were incubated with SN38 [10 nM] for the first 24 hours. Cells were then washed with PBS and incubated with eltanexor [100 nM] for an additional 48 hours. **(B)** Cell viability % measured by CellTiter Glo 2.0 and **(C)** heatmaps of the 4 CRC cell lines treated sequentially with SN38 [0 - 30 nM] followed by eltanexor [0 -100 μM]. Bliss synergy score was analyzed using SynergyFinder.

Next, we determined if there were differences between concurrent *vs*. sequential treatment of SN38 in combination with eltanexor in the HCT116, HCT15, HCT8, and LS1034 CRC cell lines. For sequential treatment, CRC cell lines were treated with SN38 for 24 hours, washed, and then treated with eltanexor for 48 hours followed by a cell viability assay at 72 hours (**Figure 4A**). In the LS1034 CRC cell line, the greatest synergy was observed at an SN38 dose of 10nM and at eltanexor doses of 0.0001 to 1 μM (**Figures 4B, C**; **Supplementary Figure 3**). In the HCT116 and HCT15 cell lines, synergy was observed with varying doses of SN38 and eltanexor with the most consistently elevated synergy score at 1 μM of eltanexor and different doses of SN38 (1, 10, and 30 nM) (**Figures 4B, C**; **Supplementary Figure 3**). We did not observe any sequential treatment effects (SN38 + eltanexor) in the HCT8 cell line at any of the doses examined. With concurrent treatment for 72 hours, there were no synergistic effects seen among all SN38 and eltanexor doses examined (**Supplementary Figure 4**). These results show that sequential treatment with the DNA damaging agent SN38 followed by eltanexor synergistically decreases CRC viability in 3 out 4 CRC cell lines, whereas no synergistic effects were observed with concurrent treatment.

### Evaluation of treatment effects on mismatch repair protein levels in CRC cell lines

**Figure 5:**
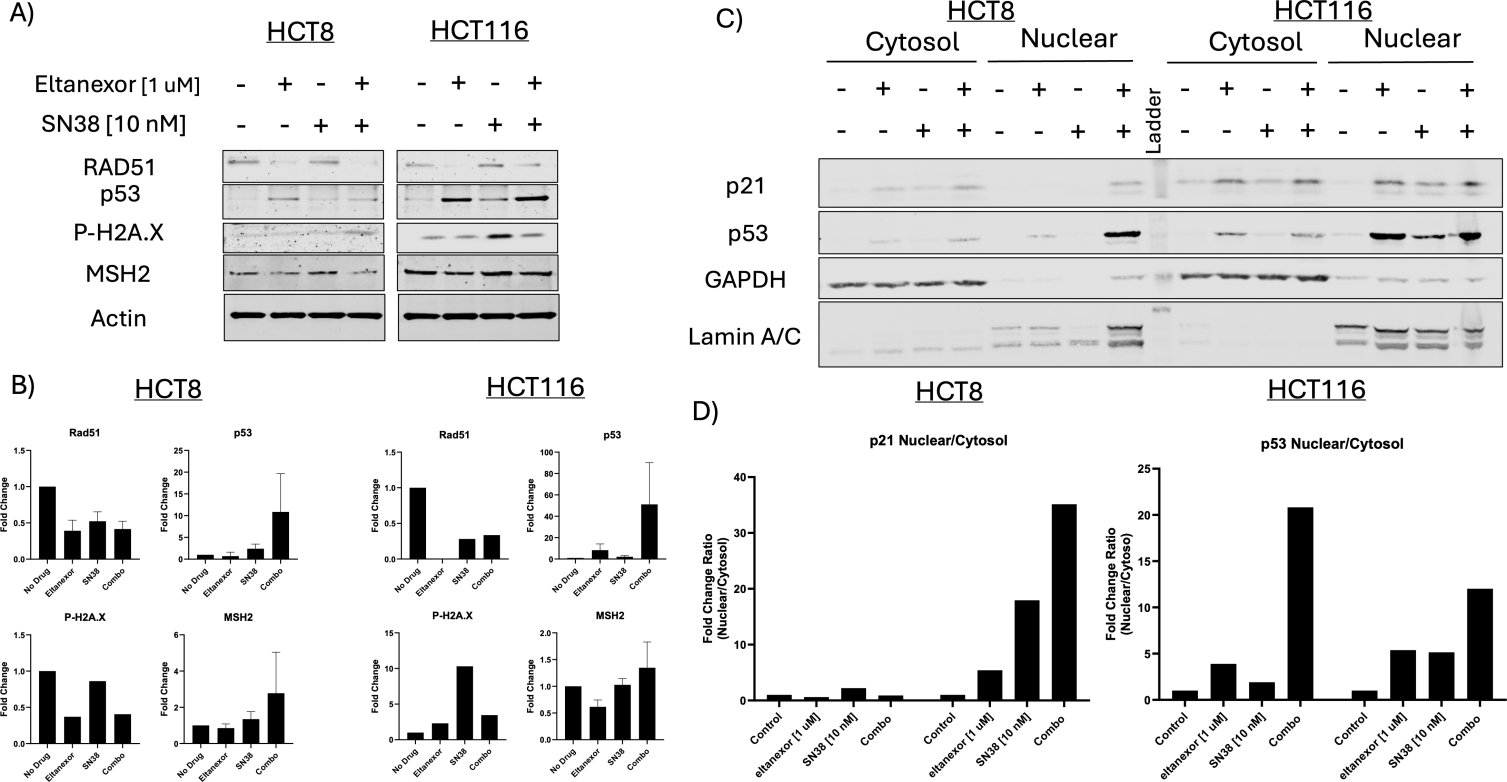
**(A)** A Western blot analysis of RAD51, p53, p-H2A.X and MSH2 in CRC cell lines HCT8, HCT116, LS1034, and HCT15 following sequential treatment of SN38 and/or eltanexor. **(B)** Densitometry analysis total cell protein. Cells were exposed to SN38 (10 nM) or vehicle for 6hr. **(C)** Cells were then washed with PBS and incubated with or without the presence of eltanexor (1 uM) for an additional 24 hr. Nuclear/Cytoplasmic. **(D)** Densitometry analysis of nuclear/cytoplasmic proteins.

In addition to determining treatment effects in our CRC254 PDX model with respect to mismatch repair genes, western blot was performed after single agent and sequential treatment in our 4 CRC cell lines. We used a dose of 10 nM for SN38 (6-hour incubation), PBS wash, and then 1 μM eltanexor (24 hours). **Figures 5A, B**, **Supplementary Figure 5** shows a decrease in the DNA damage repair protein RAD51 in the eltanexor and combination groups in HCT8 and HCT116, and in the eltanexor-treated LS1034 cell line when compared to control. A slight decrease was seen in MSH2, another DNA damage repair protein, in the HCT8 and HCT15 cell lines with eltanexor and combination, while no differences were observed in the HCT116 and LS1034 cell lines. In contrast, p53 protein was elevated in all CRC cell lines treated with SN38, eltanexor, and combination groups compared to control, with high levels seen with eltanexor and combination treatment. We also observed an increase in the phosphorylation of H2A.X, a marker of double strand DNA breaks, in LS1034 after SN38 and combination treatment, and in the HCT15 cell line after eltanexor and combination treatment. In the HCT116 cell line, activation of H2A.X was elevated after treatment with SN38. Finally, we examined nuclear and cytoplasmic protein levels of p21 and p53 in the HCT8 and HCT116 CRC cell lines. As displayed in **Figures 5C, D**, an elevation in p21 and p53 nuclear/cytoplasmic ratio was seen in the HCT116 cell line treated with eltanexor, SN38 and combination, with the greatest increase in the combination group. In the HCT8 CRC cell line, there were not many differences in the p21 nuclear/cytoplasmic ratio in all treatment groups; however, with respect to p53, the greatest increase was evident in the combination group (**Figures 5C, D**). These results demonstrate that nuclear sequestration of p53 and p21 especially in the combination group may have enhanced cellular death in the HCT116 cell line.

### Immunocytochemistry examination of the effects of sequential treatment in HCT8 and HCT116 cell lines

**Figure 6:**
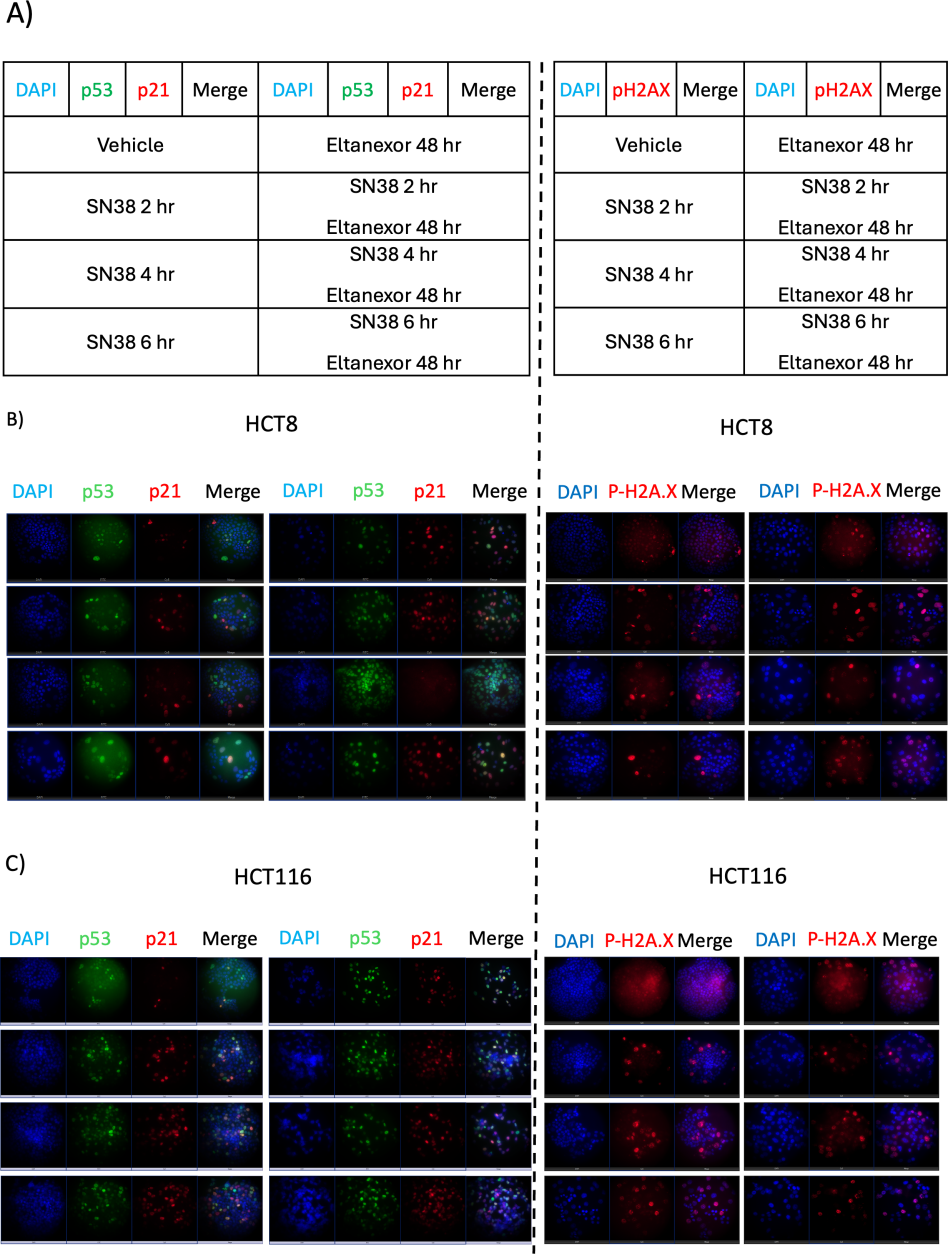
Immunocytochemistry in HCT8 and HCT 116 cell lines. Cells were first treated with SN38 (10 nM) in time series (0 hr, 2 hr, 4 hr, and 6 hr). Cells were washed with PBS and then treated with eltanexor (1 uM) for 48 hours. Cells were fixed and then stained with p53 and p21 for cell cycle arrest or stained with P-H2A.X for double stained DNA breaks. **(A)** Schematic illustration of dosing strategy, **(B)** immunostaining in HCT8 cell line and **(C)** immunostaining in HCT116 cell line.

Since we observed differences with sequential treatment in the HCT116 (synergistic decreased viability) and the HCT8 (no response) cell lines, we investigated the levels of p21, p53 and p-H2A.X using immunocytochemistry (**Figures 6A–C**). In the HCT116 cell line, levels of p21 were increased and maintained after 2 hours in the SN38 and combination groups. In the HCT8 cell line, an increase in p21 was seen in SN38 and combination, however, to a lesser extent with SN38 when compared to HCT116. Similar to the western blot analysis of p53 in the HCT116 cell line, an increase in p53 was observed by immunocytochemistry in eltanexor and combination groups after 2 hours, which was then maintained. There was a minimal difference between vehicle and SN38. The HCT8 cell line had an elevation in p53 in combination *vs*. all other groups at 2 hours and 4 hours; however, this was not maintained at 6 hours as was seen in the HCT116 cell line. p-H2A.X was increased in HCT116 at 2, 4, and 6 hours after treatment with SN38 and combination. However, in HCT8, p-H2A.X increased at 2 hours with SN38 alone and in combination, but this was not maintained at 4 and 6 hours post treatment. These results suggest that failure to maintain DNA damage and DNA damage repair changes may serve as a mechanism of resistance to combination therapy.

## Discussion

The topoisomerase inhibitor irinotecan is commonly used for the treatment of mCRC, which exerts cytotoxic effects by enhancing double strand DNA breaks. Unfortunately, tumors eventually develop resistance to irinotecan, highlighting the need for additional therapies. The nuclear export protein XPO1 plays a critical role in the transport of proteins, including TSPs and oncoproteins. Overexpression of XPO1 in mCRC may disrupt this process resulting in aberrant cellular growth. Because of this, there is increasing interest for XPO inhibition as a cancer treatment (5–7, 10, 26). Therefore, we evaluated the XPO1 inhibitor selinexor in combination with irinotecan and 5-FU in one CRC PDX model and eltanexor (which has reduced penetration through the blood brain barrier with potentially improved tolerability compared to selinexor) in combination with irinotecan in CRC cell lines and PDX models (13). We hypothesized that the addition of XPO1 inhibition may enhance irinotecan activity by altering the response to DNA damage, thereby leading to enhanced cellular death.

Using 3 CRC patient-derived PDX models, we investigated the XPO1 inhibitors selinexor or eltanexor as a single agent and in combination with chemotherapy. We first determined the treatment effects of selinexor in combination with irinotecan + 5-FU and did not observe a significant combination effect with the addition of selinexor to irinotecan + 5-FU compared with irinotecan + 5-FU. Next, we conducted a study examining a dose of 10 mg/kg eltanexor (next generation compound) and later decreased this to 7.5 mg/kg due to poor tolerability. Single agent eltanexor and single agent irinotecan did not result in significant tumor reduction. However, when combined with irinotecan, eltanexor significantly reduced tumor growth. Our second PDX study in CRC254 evaluated a lower dose of 5 mg/kg eltanexor; similar combination efficacy effects were observed, with improved tolerability. In addition, in another CRC PDX model there was single agent and combination efficacy when compared to vehicle, however no additional treatment effects were seen with combination treatment. Previous *in vivo* and *in vitro* studies have evaluated SINE monotherapy and in combination with irinotecan/SN38 in CRC cell lines and murine xenograft models, with resultant localization of topoisomerase I to the nucleus and synergistic increase in apoptosis, decreased Ki67, and inhibition of tumor growth (18, 19, 21). In a CRC xenograft model, treatment with selinexor and radiation decreased tumor growth, potentially due to DNA damage from radiation (27). Another study evaluated selinexor with the proteasome inhibitor bortezomib in the HCT116 CRC cell line, showing that the combination was effective at significantly reducing tumor growth by restoring nuclear p53 (28). The synergism of selinexor has also been seen in gastric cancer *in vivo* and *in vitro* models, with enhancement of nab-paclitaxel activity (29). In a hepatocellular carcinoma PDX model, single agent palbociclib (CDK4/6 inhibitor) or KPT-330 (XPO inhibitor) yielded modest effects on tumor burden while the combination resulted in a significant tumor burden reduction and longer survival (30). Taken together, these data demonstrate that in our preclinical CRC PDX models, differential treatment effects were evident with 1 out of the 3 CRC models showing a combinational effect with the XPO1 inhibitor eltanexor + irinotecan.

To further investigate the therapeutic potential of eltanexor and SN38 for CRC, we examined concurrent *vs*. sequential dosing in 4 CRC cell lines. While no synergistic treatment effects were observed with concurrent treatment, sequential treatment of CRC cell lines with SN38 followed by the addition of eltanexor resulted in synergy with respect to cell viability in 3 out of the 4 CRC cell lines. We did not see a synergistic treatment effect in the HCT8 which suggests that genetic heterogeneity in this cell line may have been an important factor at enhancing resistance to this combination treatment. In a previous study, pretreatment with a DNA damaging agent prior to the addition with selinexor resulted in a reduction in DNA damage repair proteins and increase cancer cell death (10). In colon cancer cell lines, the combination of the XPO inhibitor KPT-185 with SN38 worked synergistically to induce apoptosis and cell cycle arrest (18). Another study demonstrated synergy in irinotecan/SN38-resistant and -sensitive CRC cell lines with the combination of the XPO inhibitor KPT-251 and SN38 *in vitro* and *in vivo* (21). Together this suggests that the addition of a DNA damaging agent in combination with an XPO1 inhibitor may enhance cytotoxic effects and that inducing DNA damage with SN38 prior to treatment with XPO1 inhibitor may facilitate this process.

XPO1 plays a fundamental role in the in the transport of proteins, including TSPs and oncoproteins, which are key regulators of cellular function (5–7, 10, 26). Among these proteins that XPO1 transports is the tumor suppressor p53, which plays a critical role in regulating cellular responses to DNA damage by suppressing cell cycle progression and inducing apoptosis (31). In our preclinical model, we demonstrated an increase in p53 after eltanexor and combination treatment in our CRC cell lines, and this was shown to be maintained after 2 hours of treatment in the HCT116 cell line. Notably, there were only minimal differences between vehicle and SN38. We also showed an elevation in the nuclear/cytoplasmic ratio of p21 and p53 in all treatment groups in the HCT116 cell line with the greatest increase observed in the combination group. Preclinical studies in CRC and other solid malignancies have shown that XPO1 inhibition enhanced p53 protein levels and induced cell cycle arrest and apoptosis (14, 15, 17). Given that we observed synergistic effects in the HCT116 cell line with SN38 + eltanexor with an increase in total p53 and nuclear p53 levels, it is likely that combination treatment prevented nuclear export of p53, resulting in induction of apoptosis and anti-tumor efficacy. In addition to p53, in response to DNA damage, H2A.X is phosphorylated and is an important mediator in regulating the cell cycle. We showed that in combination, the activation of H2A.X was increased and maintained for 6 hours in the sensitive HCT116 cell line. This is consistent with a prior study using osteosarcoma cells which showed that DNA damage (γH2A.X) due to doxorubicin persisted longer when followed by selinexor *vs*. doxorubicin alone, altering the cells’ ability to repair DNA damage after treatment with doxorubicin (10). In our CRC PDX model, we did not see as much difference in the expression in DNA damaging pathway genes, and this may be a result of timing and is a limitation in this study as we measured gene expression only at day 7. These results support that XPO1 inhibition may be effective when given after a DNA damaging agent, allowing DNA damage to persist. However, further mechanistic work is needed to determine this in our preclinical models.

Interestingly, in our CRC PDX model we observed a significant increase in the gene expression of *GUSB* with combination treatment. GUSB is an enzyme that functions in degrading glycosaminoglycans in the lysosome (32). Cellular stress from irinotecan and/or alterations in protein trafficking by XPO1 inhibition may have played a role in enhancing the expression of GUSB. Additional studies to better understand the mechanistic role of GUSB in drug responses to eltanexor and irinotecan are needed. Another potential mechanism whereby drug resistance to irinotecan may occur is through the export of topoisomerases out the nucleus by XPO1 (21, 33, 34). A study by Chung et al. showed in a preclinical model of CRC that the inhibition of XPO1 with KPT-251 increased topoisomerase I in the nucleus, potentially sensitizing these cells to irinotecan treatment (21). A study in acute myeloid leukemia (AML) investigating the combination of selinexor with topoisomerase II inhibitors revealed an increase in topoisomerase II and a synergistic combinational effect in AML cell lines (34). Preventing efflux of topoisomerase I out of the nucleus via XPO1 inhibition may further enhance irinotecan effects and result in an increase in sensitivity to irinotecan. Both mechanisms need to be explored to better understand combinational relationship whereby eltanexor enhances the effects of irinotecan.

In addition, we observed toxicity in mice with higher doses of eltanexor (initially 10 mg/kg followed by 7.5 mg/kg) and irinotecan. We subsequently reduced the dose of eltanexor to 5 mg/kg. The toxicity observed is consistent with previous clinical experience with XPO1 inhibitors, as seen in the phase I dose-escalation trial combining selinexor with chemotherapy in patients with mCRC (22). Patients received mFOLFOX6 plus selinexor (on days 1, 3, and 8 every 14 days) in dose-escalation. All 4 patients treated with selinexor 40 mg experienced dose-limiting toxicities. The most common grade 3 or higher adverse events were gastrointestinal symptoms (30% nausea, 20% vomiting, and 10% diarrhea). Due to short treatment exposure from toxicities, efficacy could not be assessed. In a phase 1 trial for patients with relapsed/refractory multiple myeloma, 122 patients were treated with selinexor (35). Prophylactic antiemetic ondansetron was required prior to selinexor administration. Dose-modifications were common and occurred in 80% of the study population, with most adverse events occurring in the first 2 cycles. While eltanexor was thought be better tolerated given the decreased blood brain barrier penetration, these adverse events in our study may represent the need for further dose-reduction or change in dosing frequency. In the phase 1 trial of eltanexor in patients with relapsed/refractory multiple myeloma, adverse events were consistent with selinexor (thrombocytopenia, neutropenia, anemia), though the incidence and severity of nausea, decreased appetite, and fatigue were lower. Platelet transfusions and thrombopoietin receptor agonists were permittable. Again, prophylactic antiemetics were utilized and dose reductions and interruptions were common (38% and 72%, respectively). Additional studies in our preclinical model are needed to evaluate different dosing of eltanexor in combination with irinotecan with the goal of maximizing efficacy while limiting toxicity.

Phase I XPO1 inhibitor CRC clinical trial results have been disappointing, with limited efficacy observed with single agent selinexor and intolerability of combination selinexor with FOLFOX. Whether there is clinical efficacy with the more tolerable SINE eltanexor in combination with irinotecan, potentially with sequential treatment, to treat mCRC is not known. Should additional preclinical studies show improved tolerability with optimal dosing, sequential treatment with XPO1 inhibition and irinotecan may be studied in the future.

## Conclusion

Overall, we demonstrated the therapeutic efficacy of combination XPO1 inhibitor, eltanexor, and irinotecan in a subset of our CRC PDX and CRC cell line preclinical models. Sequential treatment with a DNA damaging agent first, then XPO1 inhibitor, may be most effective at enhancing antitumor effects. Further preclinical studies are needed to improve tolerability of these compounds and to have a better understanding of the differences in anti-tumor efficacy between responders and non-responders to this combination.

## Data Availability

The original contributions presented in the study are included in the article/**Supplementary Material**. Further inquiries can be directed to the corresponding author.

## Supplementary material

Supplementary Figure 1 Net body weight percent change in the *in vivo* PDX CRC254, BPB56DDD, and CRC238 studies. **(A)** CRC254 first study average per group, corresponding to **Figures 1A, B**, and **(B)** Second CRC254 study average per group, corresponding to **Figures 1E, F**, **(C, D)** CRC254 individual mice percent change in net body weight. **(E, F)** First CRC254 study percent change in net body weights individually and average per group. **(G)** BPB56DDD study average per group corresponding to **(H)** individual net body weight per group. **(I)** CRC238 study average per group corresponding to **(J)** individual net body weight per group.

Supplementary Figure 2 Patient derived xenograft (PDX) tumor CRC238 treated with single agent eltanexor, irinotecan, or combination. **(A, B)** Assessment of tumor volumes **(A)** and specific growth rate (SGR) **(B)** after 23 days of treatment.

Supplementary Figure 3 3D synergy plot of CRC cell line viability with sequential dosing in HCT8, HCT116, LS1034, and HCT15.

Supplementary Figure 4 Cell line viability of CRC cell lines HCT8, HCT116, LS1034, and HCT15 with concurrent dosing.

Supplementary Figure 5 Westerns of LS1034 and HCT15.
