# Supplementary material for: Efficacy of an XPO1 inhibitor in combination with irinotecan in a preclinical colorectal cancer model
Source: Front Oncol. 2026 Mar 31;16:1721685. doi: 10.3389/fonc.2026.1721685 (PMC13076135; doi:10.3389/fonc.2026.1721685)
Supplement: Supplementary Figure 1 — Net body weight percent change in the in vivo PDX CRC254, BPB56DDD, and CRC238 studies. (A) CRC254 first study average per group, corresponding to Figures 1A, B, and (B) Second CRC254 study average per group, corresponding to Figures 1E, F, (C, D) CRC254 individual mice percent change in net body weight. (E, F) First CRC254 study percent change in net body weights individually and average per group. (G) BPB56DDD study average per group corresponding to (H) individual net body weight per group. (I) CRC238 study average per group corresponding to (J) individual net body weight per group. [file Presentation1.pptx]

## Slide 1
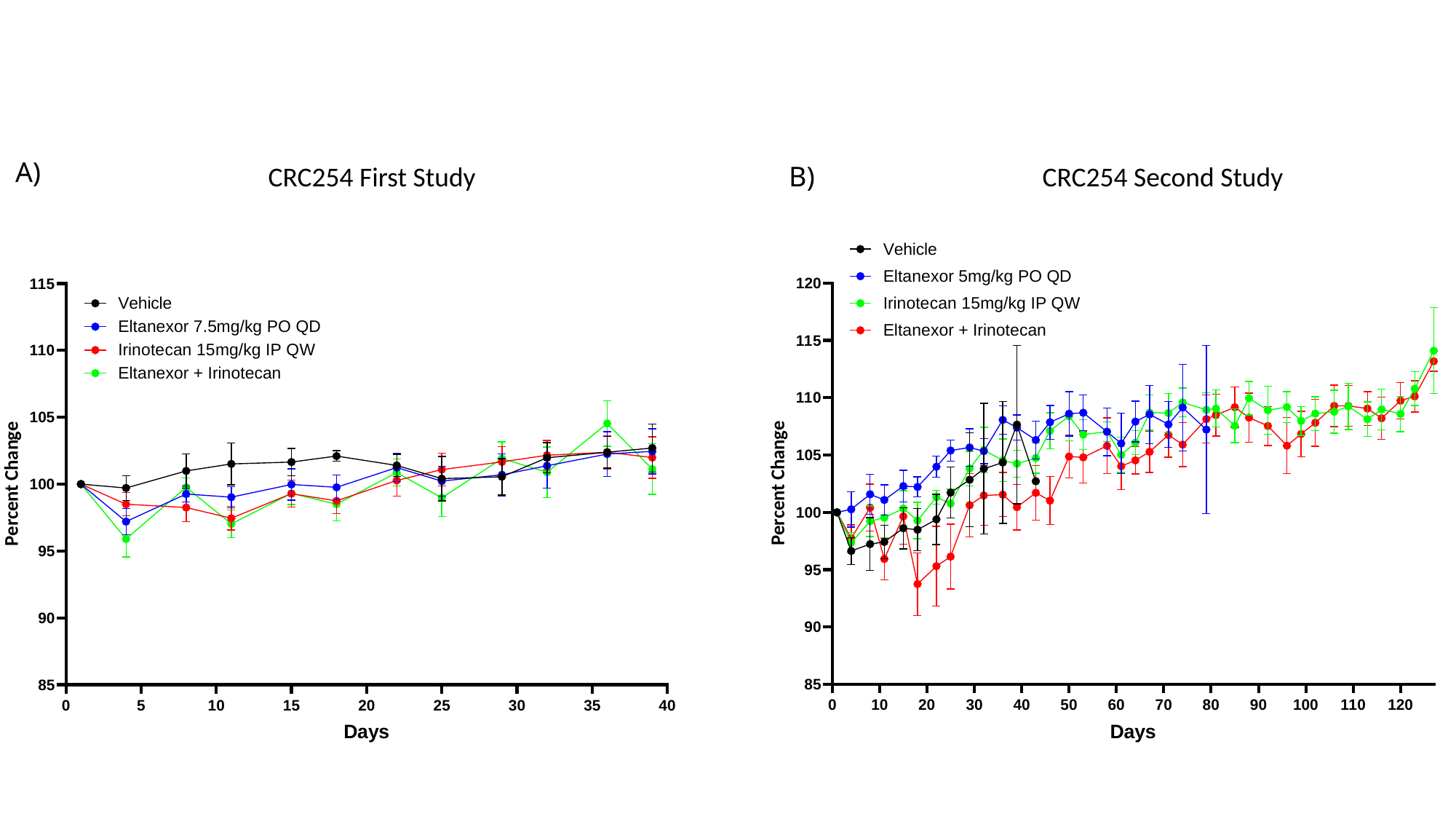

A)
CRC254 First Study
B)
CRC254 Second Study

## Slide 2
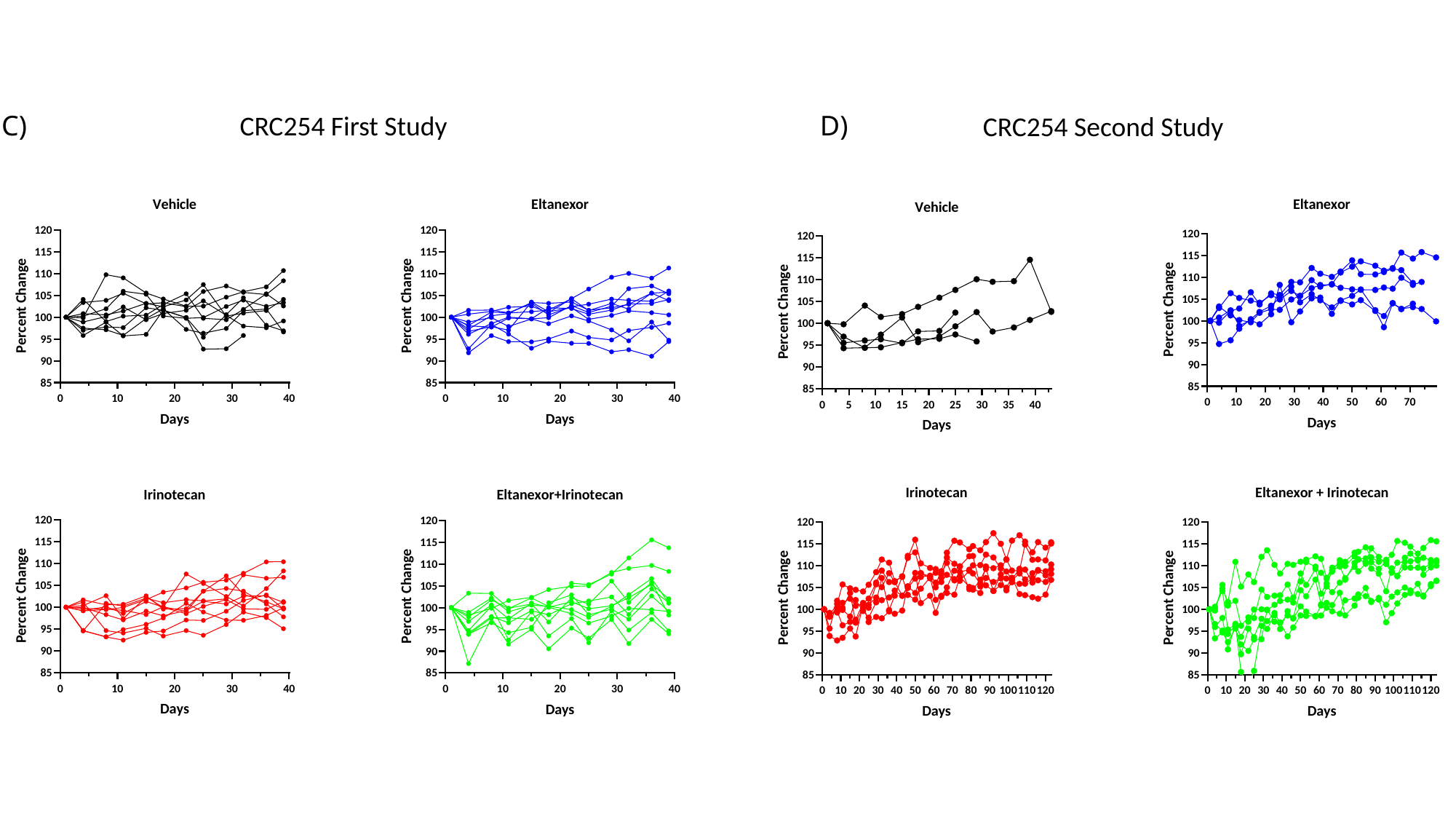

C)
CRC254 First Study
D)
CRC254 Second Study

## Slide 3
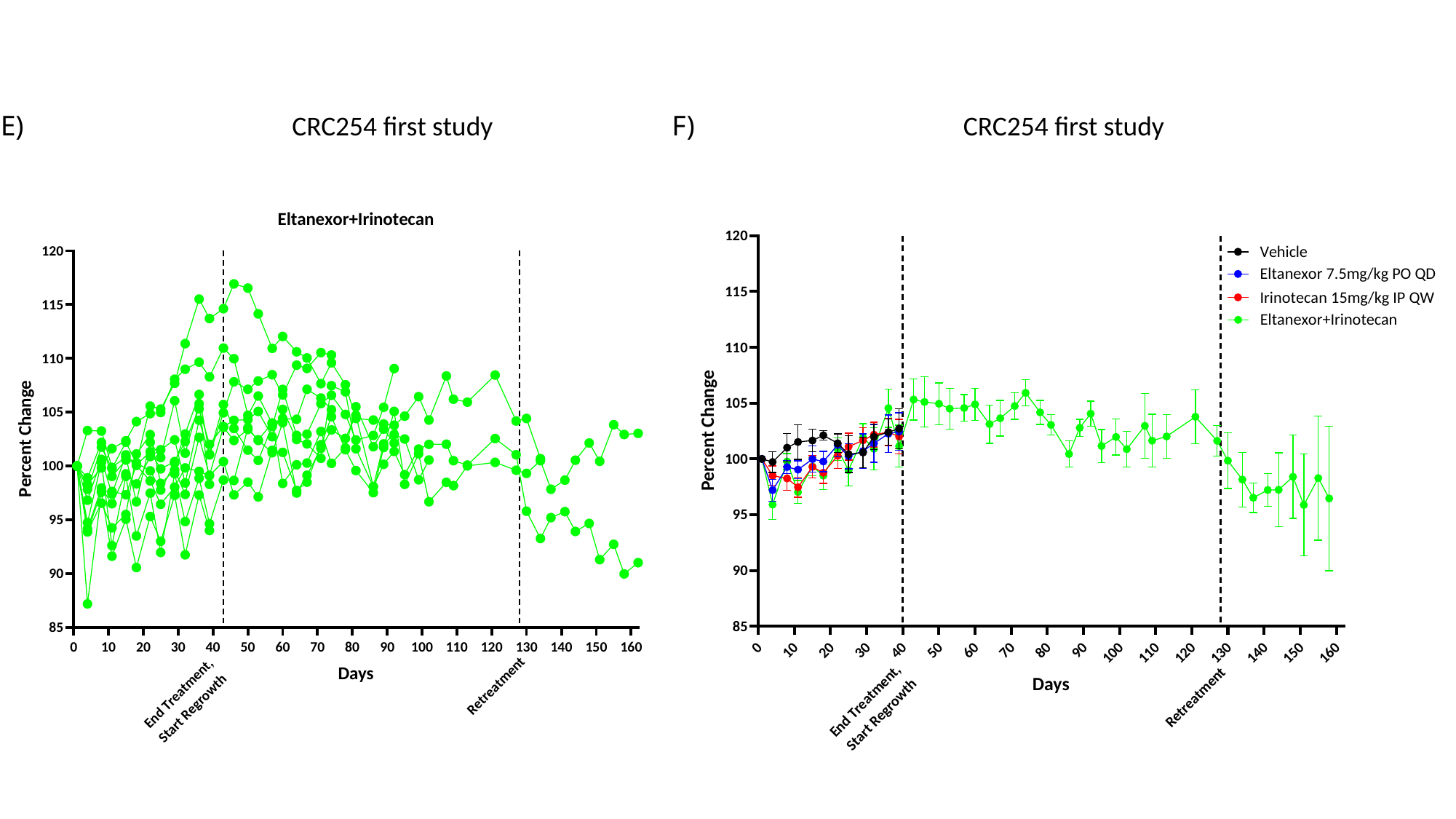

E)
CRC254 first study
F)
CRC254 first study

## Slide 4
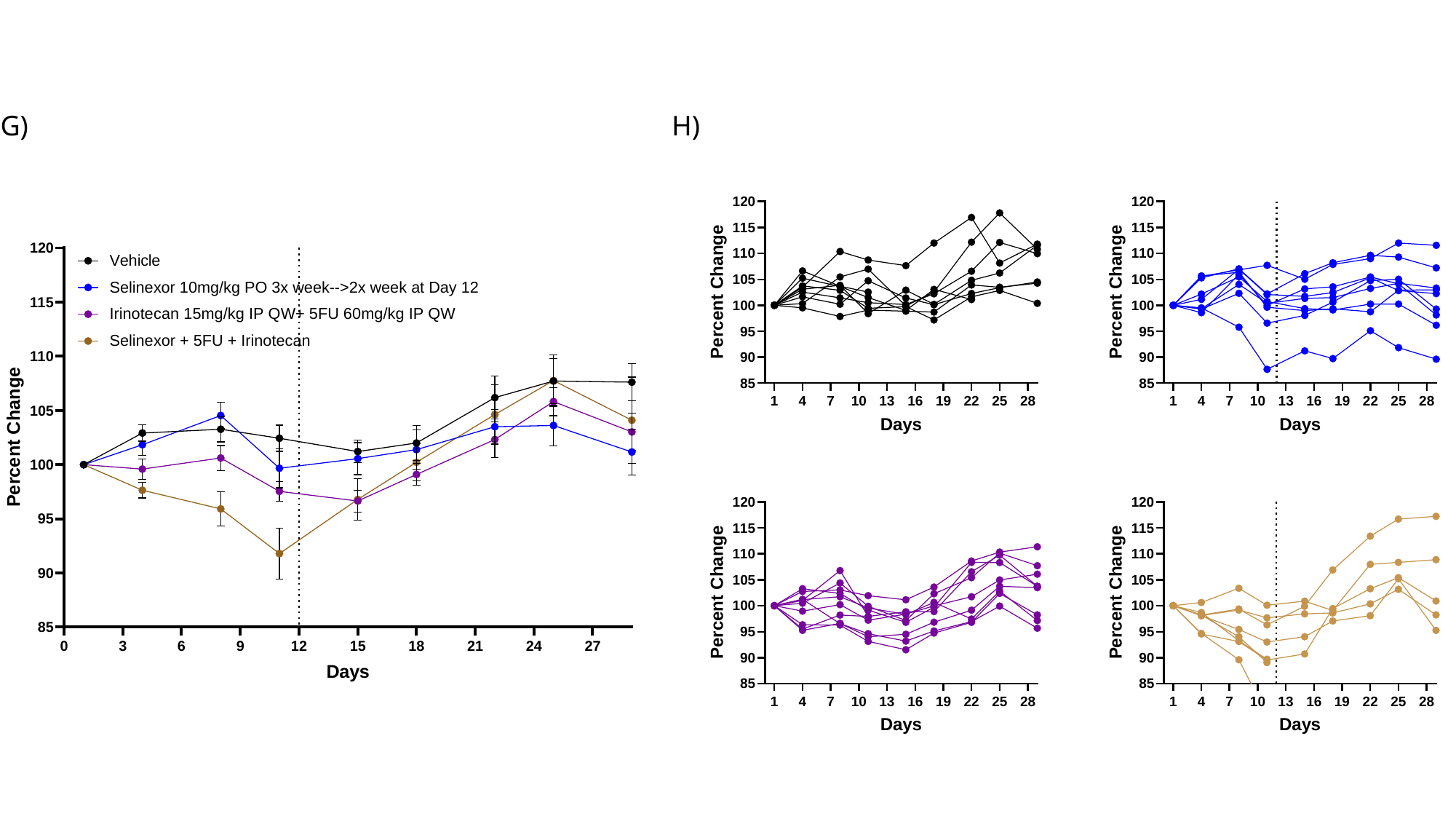

G)
H)

## Slide 5
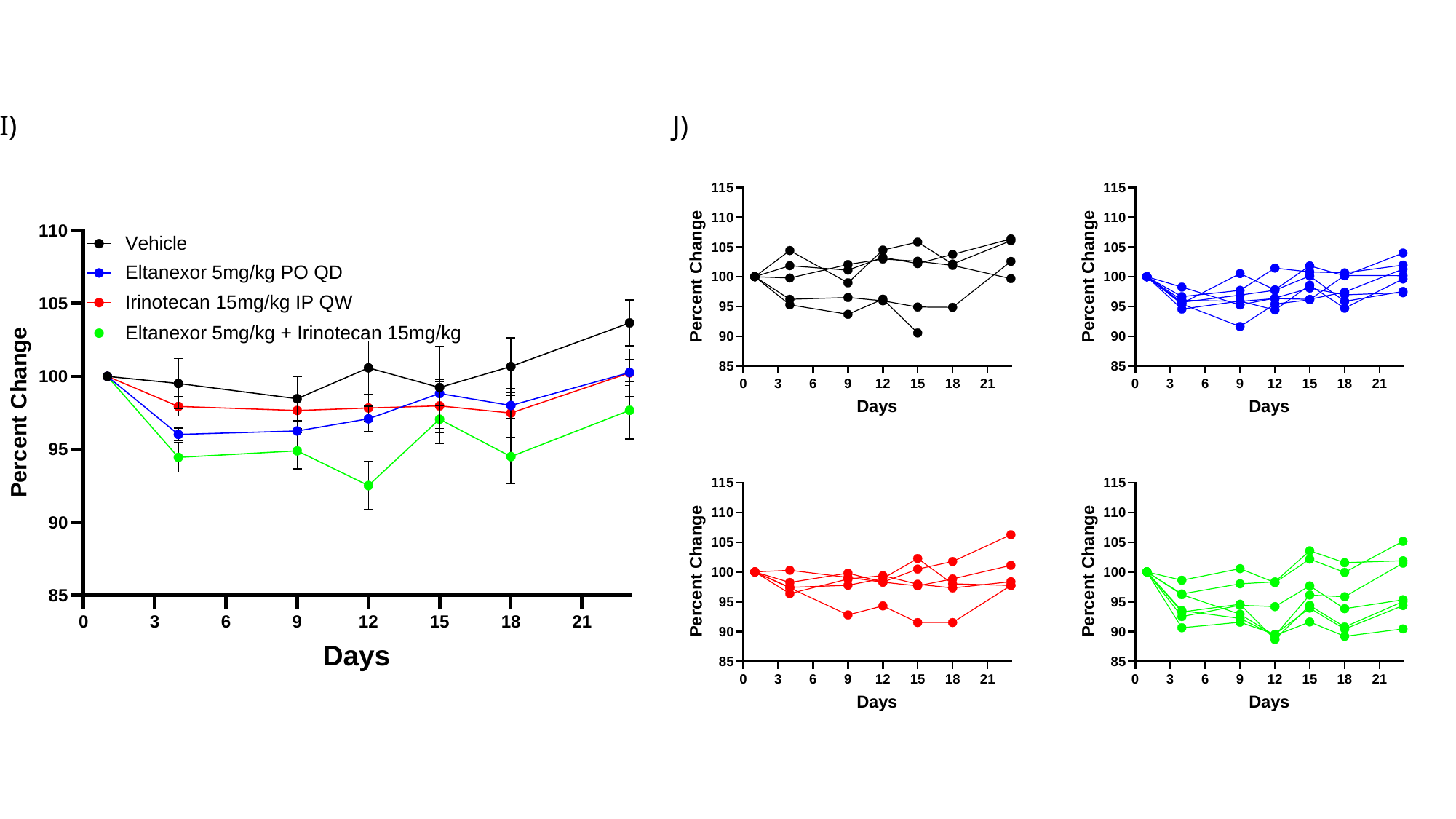

I)
J)
