# Supplementary figures and images for: Efficacy of an XPO1 inhibitor in combination with irinotecan in a preclinical colorectal cancer model
Source: Front Oncol. 2026 Mar 31;16:1721685. doi: 10.3389/fonc.2026.1721685 (PMC13076135; doi:10.3389/fonc.2026.1721685)

## Slide 1
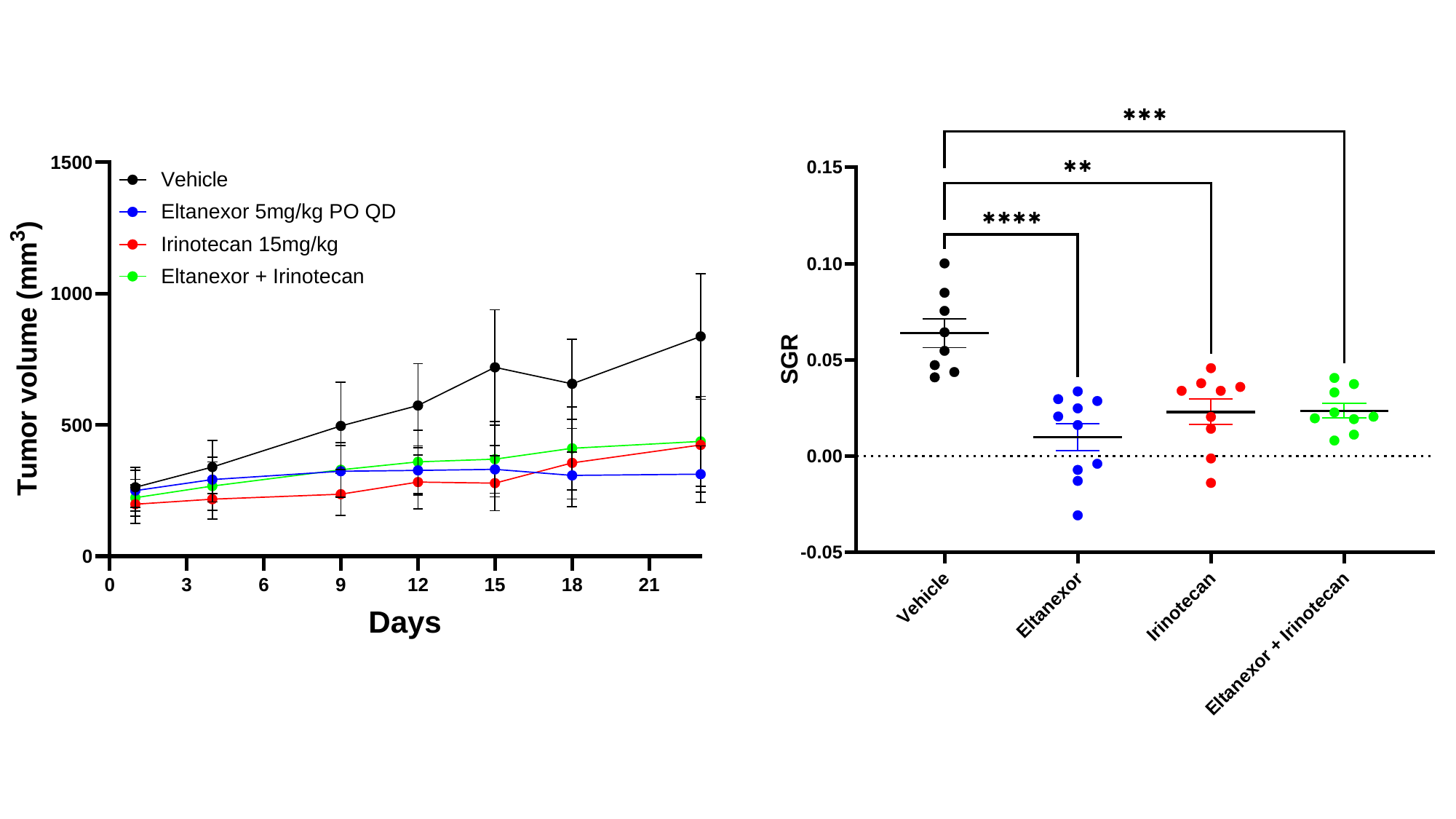

Supplement: Supplementary Figure 2 — Patient derived xenograft (PDX) tumor CRC238 treated with single agent eltanexor, irinotecan, or combination. (A, B) Assessment of tumor volumes (A) and specific growth rate (SGR) (B) after 23 days of treatment. [file Presentation2.pptx]

## Slide 1
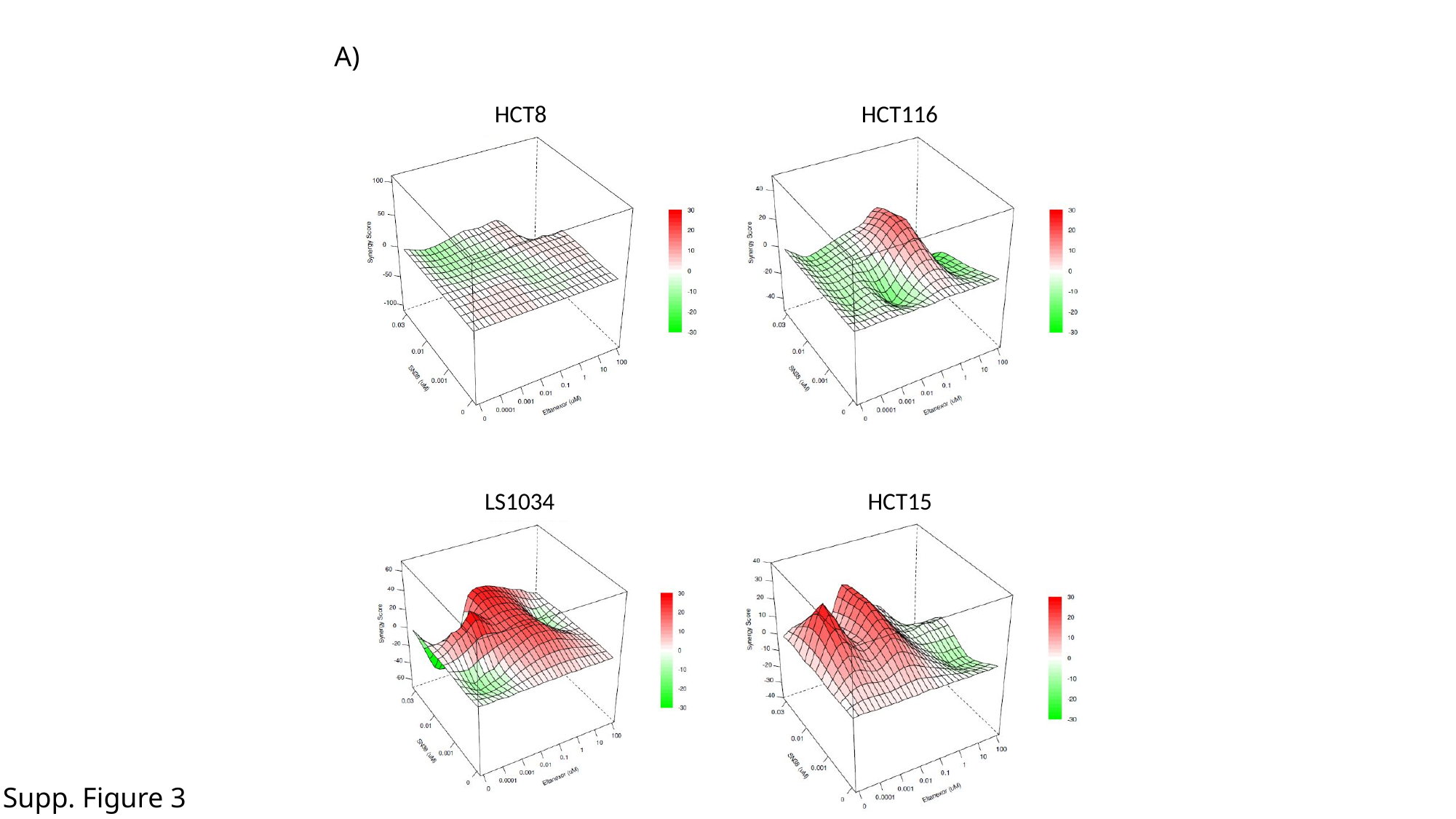

A)
HCT116
HCT8
HCT15
LS1034
Supp. Figure 3

Supplement: Supplementary Figure 3 — 3D synergy plot of CRC cell line viability with sequential dosing in HCT8, HCT116, LS1034, and HCT15. [file Presentation3.pptx]

## Slide 1
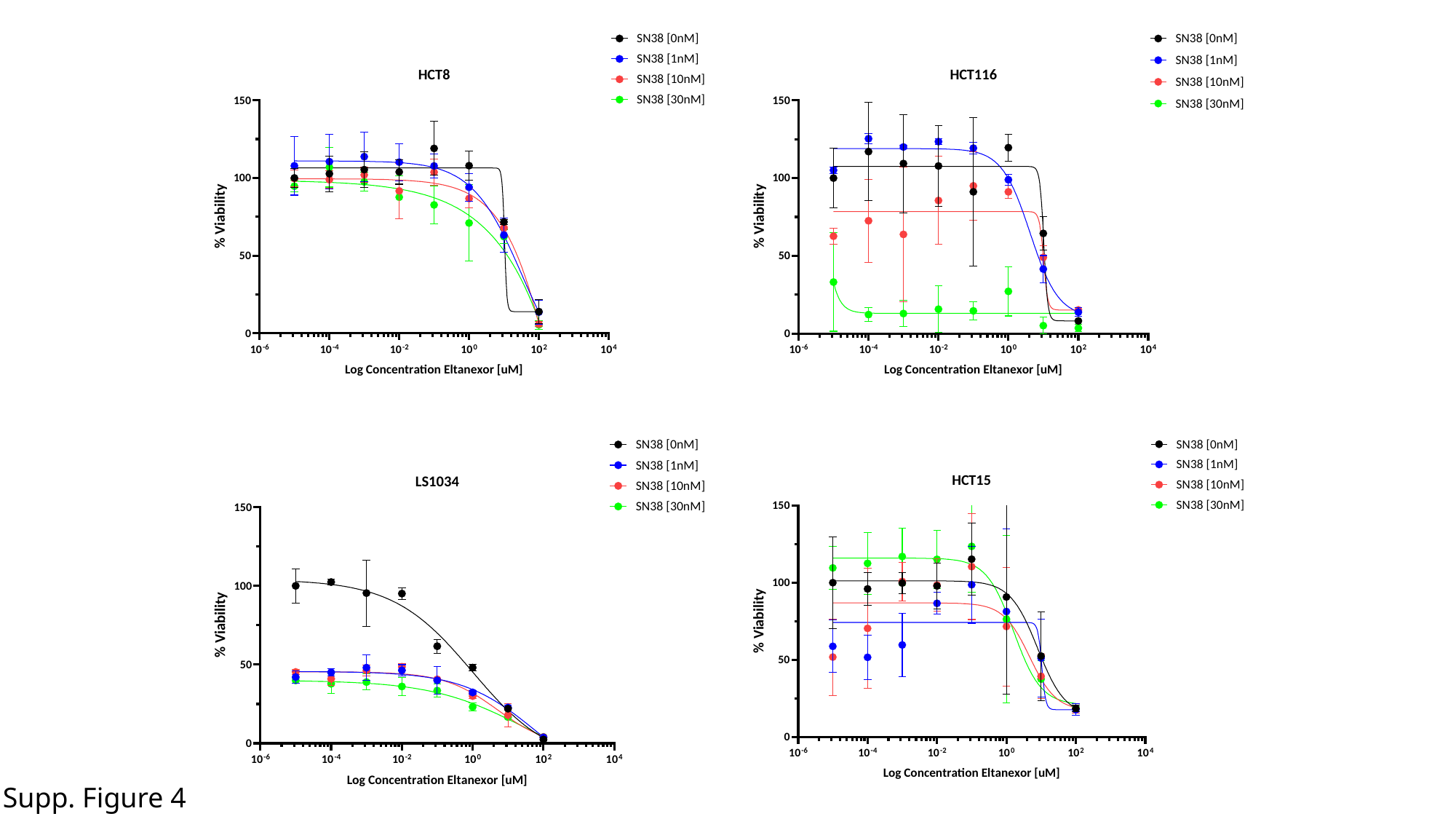

Supp. Figure 4

Supplement: Supplementary Figure 4 — Cell line viability of CRC cell lines HCT8, HCT116, LS1034, and HCT15 with concurrent dosing. [file Presentation4.pptx]

## Slide 1
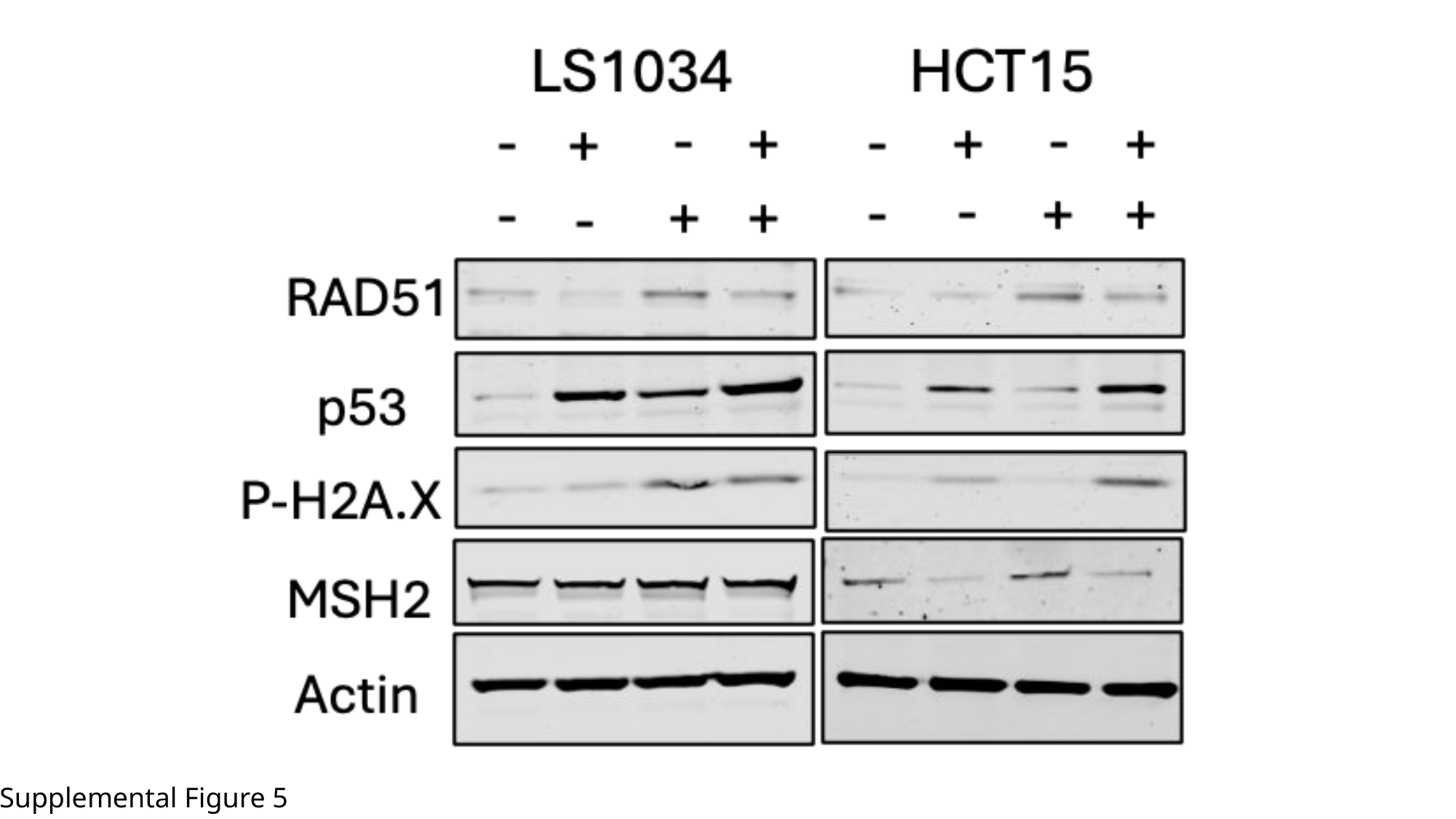

Supplemental Figure 5

Supplement: Supplementary Figure 5 — Westerns of LS1034 and HCT15. [file Presentation5.pptx]
